# Reduced prefrontal activity and suicidal behaviour in early schizophrenia

**DOI:** 10.1192/j.eurpsy.2021.1449

**Published:** 2021-08-13

**Authors:** M. Grigoriou, R. Reniers, P. Mallikarjun, R. Upthegrove

**Affiliations:** 1 Institute For Mental Health, University of Birmingham, BIRMINGHAM, United Kingdom; 2 Institute For Mental Health, University of Birmingham, Birmingham, United Kingdom

**Keywords:** suicidal behaviour, schizophrénia, FEP, prefrontal activity

## Abstract

**Introduction:**

Approximately, 15- 26% of patients with first-episode psychosis, including schizophrenia, are likely to have attempted suicide by their first treatment contact. Studies of suicidal behavior outside of schizophrenia have indicated grey matter volume loss in the prefrontal and orbitofrontal cortex, and aberrant brain activity in relation to emotional recognition and dysfunction.

**Objectives:**

This study aimed to investigate the functional neural correlates of suicidal behavior in early schizophrenia.

**Methods:**

fMRI faces task was conducted (fearful face versus neutral face) in 8 participants with first-episode schizophrenia together with standardised scales including PANSS and SBQ-R. fMRI activation was compared using a two-sample t-test in participants with low and high suicidal behavior. Extent threshold is 0 voxels and significance level p<0.001 (FWE corrected). Processing of images was carried out using SPM12 and Matlab.

**Results:**

8 participants were recruited; 5 males and 3 females, mean age of 26.5. Results suggest that participants with higher suicidal behaviour showed reduced activation on the anterior-cingulate gyrus and medial frontal gyrus, which are parts of PFC, (p= .005). There was also a significant difference in task response accuracy, where, participants with high suicidal behaviour made more accurate responses compared to low group (t (3) = 3.65, p = .035).

**Conclusions:**

This is an exploratory study, investigated the differences in brain activity in patients with schizophrenia who are at risk of completed suicide and, therefore might provide new insights into the underlying mechanisms. Further work should address how PFC activity changes with risk over time and its potential utility as a biomarker in suicide.

